# Emerging Role of Follicular T Helper Cells in Multiple Sclerosis and Experimental Autoimmune Encephalomyelitis

**DOI:** 10.3390/ijms19103233

**Published:** 2018-10-19

**Authors:** James L. Quinn, Robert C. Axtell

**Affiliations:** 1 Department of Microbiology and Immunology, University of Oklahoma Health Sciences Center, Oklahoma City, OK 73104, USA; 2 Arthritis and Clinical Immunology Program, Oklahoma Medical Research Foundation, Oklahoma City, OK 73104, USA

**Keywords:** TFH, B-cells, multiple sclerosis, EAE, TH17

## Abstract

Multiple sclerosis (MS) is an autoimmune disorder where both T cells and B cells are implicated in pathology. However, it remains unclear how these two distinct populations cooperate to drive disease. There is ample evidence from studies in both MS patients and mouse models that Th17, B cells, and follicular T helper (TFH) cells contribute to disease. This review article describes the literature that identifies mechanisms by which Th17, TFH, and B cells cooperatively drive disease activity in MS and experimental autoimmune encephalomyelitis (EAE). The curation of this literature has identified that central nervous system (CNS) infiltrating TFH cells act with TH17 cell to contribute to an inflammatory B cell response in neuroinflammation. This demonstrates that TFH cells and their products are promising targets for therapies in MS.

## 1. Introduction

Multiple sclerosis (MS) is a chronic autoimmune disorder that is characterized by inflammation in the central nervous system (CNS) and demyelination of neurons and leads to collateral damage in neighboring CNS tissue [1]. It is estimated that MS affects approximately 2.5 million people worldwide. While the cause of MS is not known, strong evidence suggests genetic and environmental factors are involved in the initiation of this disease [2]. The majority of MS patients (85%) have episodes of disability followed by almost full recovery. As such, they are categorized as having a relapsing-remitting phenotype (RRMS). Over time, approximately 50% of RRMS patients will develop the secondary progressive version of this disease (SPMS), where inflammatory episodes decrease, but permanent neurological damage continues to develop [3]. Even though there is no cure for this disease, there are 9 classes of Food and Drug Administration (FDA) approved drugs that effectively reduce inflammatory activity in MS and only 1 that has been approved for progressive versions of this disease [4]. 

Over many decades, accumulating data show that CD4+ T helper (Th) cells, particularly Th1 and Th17 cells, play a critical role in inducing the inflammatory pathology of this disease. CD4+ cells are observed in the lesions of MS patients, polymorphisms in the MHCII genes are major genetic risk factors for disease [5], and MS-like disease, called experimental autoimmune encephalomyelitis (EAE), can be induced in mice through the transfer of autoreactive T cells into healthy recipient animals [6]. Despite this important role for T cells, more recent studies have found that B-cells also significantly contribute to disease. In fact, recent exciting clinical trial results have led to the FDA approval of the B-cell-depleting antibody, ocrelizumab, for MS [7,8].

These observations described above demonstrate that both CD4+ T cells and B cells have critical roles in MS pathology, but it remains unclear how these cells cooperate to promote disease activity and progression in MS patients. One potential connection between these two populations is follicular T helper cells (TFH). This recently identified CD4+ subset has been shown to have potent activity in driving B-cell responses and recent studies have now begun to unravel their particular role in MS and in EAE. This review will discuss how these TFH cells act as a critical link between inflammatory Th17 cells and pathogenic B-cells, and how this axis can contribute to disease in MS patients.

## 2. Follicular T Helper (TFH) Cells

While it has been well known that CD4+ T cells are capable of promoting B-cell activity, it was not until 2008 that the distinct follicular T helper (TFH) cells were identified as a unique lineage of helper T cells. TFH cells were initially described as a subset of the CD4+ T cell population that expressed the chemokine receptor CXCR5, were found in the B-cell follicles in secondary lymphoid tissues, and were extremely effective in driving germinal center formation and inducing B-cell response [9,10,11]. After BCL6 was identified as the master transcription regulator of these CXCR5+ CD4+ cells, TFH cell are now recognized as an independent and distinct T cell subset [12,13,14]. Since their discovery, TFH cells have been shown to be critical for effective B-cell activity in many different models of infection and vaccination. These cells have also been shown to be associated with many different autoimmune diseases including systemic lupus erythematosus (SLE) [15], rheumatoid arthritis (RA) [16], and MS [17].

Currently, there is not a complete and thorough understanding of how naïve CD4+ helper T cells differentiate into mature TFH cells. However, many studies have provided some inroads on to the mechanisms involved in TFH differentiation. These studies have indicated that proper differentiation is “multistage and multifactorial” [18,19]. The cytokines IL-6 and IL-21 are important for effective differentiation [20,21]. IL-6 signaling through STAT3 and STAT1 leads to BCL6 expression in T cells in vitro [22] and IL-6 leads to IL-21 production. IL-21 can also lead to BCL6 expression in T cells though there appears to be substantial overlap in signaling between these cytokines [21]. Further complicating the process, experiments in knockout mice demonstrate that they are not the only factor involved [23,24]. There is also evidence that the quality and duration of TCR/MHCII interaction during initial stimulation can affect whether a naïve T cell will differentiate into a TFH cell. Certain models demonstrate that T cells with a high affinity TCR are more likely to differentiate into TFH cells [25]. 

Finally, complete differentiation of early TFH cells into mature TFH cells is dependent on interactions with B-cells [19]. Sustained T cell/B-cell interactions are partially achieved through ICOS-ICOSL. Mice which lack ICOS have a sufficient deficiency in their TFH population [26]. Interestingly, one study demonstrated that ICOSL expression on the noncognate, bystander B-cells serves to keep T cells within the germinal center (GC) and allow for their proper differentiation into TFH cells [27]. Although B-cells clearly are important for proper TFH differentiation, this differentiation process can still occur in the absence of B-cells under certain conditions [28]. All of these studies together demonstrate that there is a positive feedback loop between B-cells and TFH cells which is in part due to their similar expression of BCL6 and their reliance on similar cytokines for proliferation and differentiation. These cytokines include IL-6, IL-21, and BAFF; all of which signal on both TFH and B-cells. It is often a “chicken or egg” situation when examining whether a germinal center deficiency is a result of direct TFH inhibition or inhibition of B-cells leading to TFH inhibition. The important concept to keep in mind is that both populations stimulate and promote each other in vivo.

### 2.1. Functions of TFH Cells

TFH cells were initially identified in human tonsils in close proximity to B-cells [10]. It has since been shown that TFH cells are able to localize to the B-cell follicle via expression of CXCR5 and downregulation of CCR7. Once in the follicle, TFH cells can provide help to B-cells by supporting their survival, proliferation, differentiation, and expansion. This requires a number of distinct roles for the TFH cells within the germinal center. They must be able to initiate the formation of the GC, induce somatic hypermutation in the B-cells, keep proliferating B cells alive and select for the most successful B cell receptor (BCR) mutations, induce class-switching, and stimulate their differentiation into memory B-cells and plasma cells. These diverse, and often contradicting, roles are achieved by TFH cells using a wide array of signals at different stages throughout the process. Due to this complexity, many of the mechanisms involved in these processes remain unelucidated. 

The principal B-cell helper mechanism of TFH cells is through cytokine production. The predominant cytokine produced by TFH cells is IL-21. This cytokine has been shown to promote plasma cell differentiation in both humans and mice [23,29]. Others have demonstrated that TFH cells also secrete other B-cell-associated cytokines such as IL-4 [30] and BAFF [31], as well as the chemokine CXCL13 [32]. 

Apart from cytokines and chemokines, TFH cells can provide survival signals to proliferating B-cells through receptors such as CD40L and the TCR [19]. Inversely, GC TFH cells also express high levels of FasL on their surface. This allows the TFH cells to induce apoptosis in the Fas-expressing B-cells within the germinal center. B-cells undergoing somatic hypermutation are able to escape death by receiving BCR-induced survival signals that overcome this Fas-FasL apoptosis signal. The B-cells with low affinity BCRs fail to receive these survival signals and undergo apoptosis. Once successful and productive somatic hypermutation has occurred, surviving B-cells are able to develop into memory B-cells or plasma cells and leave the germinal center [33].

Another critical receptor highly expressed on TFH cells is PD-1. This receptor is responsible for inhibiting T cell proliferation and is associated with T cell exhaustion and tumor microenvironments [34]. In the germinal center where TFH cells are constantly experiencing TCR stimulation, PD-1 helps prevent these TFH cells from proliferating out of control. Therefore, knocking out this signaling results in more TFH cells in the germinal center, but worse B-cell responses due to a less competitive environment [35]. 

The processes of class-switching and somatic hypermutation, which both require activation-induced cytidine deaminase (AID), seem to be linked to TFH cell function, although the exact signaling and transcription mechanisms remain unclear. Similarly, the differentiation of plasma cells and memory cells and their exit from the GC has links to TFH activity, particularly IL-21 signaling leading to plasma cell formation [36], but recent evidence suggests there are also inherent properties of B-cells driving the development of memory cells [37]. 

### 2.2. Subsets of TFH Cells in Humans

Outside of germinal centers in secondary lymphoid tissue, TFH cells have been observed circulating in the blood in both humans and mice. These CXCR5+ cells were originally difficult to identify for several reasons. First, all T-cells transiently express CXCR5 upon initial T cell receptor (TCR) activation [38]. Therefore, it was believed that these blood CXCR5+ T cells were simply activated T cells. The second issue with identifying these circulating TFH cells is their lack of BCL6 expression [39,40,41,42,43]. This is particularly surprising because BCL6 is necessary for TFH differentiation [14]. However, more recent studies found that these cells lacked other traditional activation markers and maintained CXCR5 expression for long periods, even in the absence of activation [44]. Moreover, these CXCR5+ T cells were more potent in activating B-cell responses in cell culture experiments compared to the CXCR5-T effector population [41]. Finally, the lack of BCL6 expression on these circulating TFH cells are thought to reflect the ability of TFH cells to downregulate BCL6 following commitment to TFH differentiation. 

Additionally, these circulating TFH cells have been shown to be quite heterogeneous and have a range of subsets corresponding to the traditional CD4+ effector T cell subsets. These subsets can be identified by their expression of chemokine receptors CCR6 and CXCR3 and transcription factors. The Th1-like TFH subset is CXCR3+CCR6-Tbet+, the Th2-like TFH subset is CXCR3-CCR6-GATA3+, and the Th17-like TFH subset is CXCR3-CCR6+RORγT+. Each subset secretes both IL-21 as well as the cytokines associated with their corresponding subset; IFNγ, IL-4 and IL-13, and IL-17 respectively [39]. Others have found that these subsets of circulating TFH cells have different abilities to act on B-cells and promote antibody production with Th17- and Th2-like TFH cells able to induce ample antibody production while TH1-like TFH cells were unable to promote this B-cell stimulation [39,45]. 

Apart from circulating TFH subsets, regulatory TFH (Tfr) cells have also been recently discovered in humans. As a subset of regulatory T cells, these Tfr cells can be identified by their expression of CXCR5 and BCL6, as well as FOXP3 [46]. These cells are thought to primarily act within the germinal center and serve to inhibit T cell-B-cells interactions, resulting in the reduction of affinity maturation and antibody production [47].

## 3. B-Cells in Multiple Sclerosis (MS)

Although MS was originally thought to be primarily mediated by CD4+ T cells based on their presence in the CNS, B-cells have recently been shown to play a critical role in this disease. In particular, the B-cell-depleting anti-CD20 antibodies therapies (ocrelizumab and rituximab) have been effective in treating MS patients [7,48]. One of the earliest indications that there is a humoral component in this disease is the presence of oligoclonal banding in the cerebrospinal fluid of MS patients. This banding indicates that antibody-secreting cells are present in the CNS during disease and they are producing antibodies to specific epitopes [49]. However, the precise role of autoantibodies in MS remains controversial for several reasons. First, it is not clear what antigens are being targeted by the antibodies. Second, anti-CD20 is effective in MS patients despite not targeting antibody-producing plasma cells, which do not express CD20. Instead, antibody levels are not significantly reduced in patients following B-cell-depleting therapy [8,48]. Nevertheless, there is evidence that autoantibodies contribute to disease to some degree because plasmapheresis effectively treats an active MS relapse and can be effective therapy in a subset of patients [50,51].

Although autoantibodies may be a factor in disease progression, it appears that B-cells are contributing to MS disease pathology through additional mechanisms. One of these mechanisms is the production of pro-inflammatory cytokines. Studies have shown that inflammatory B-cells can secrete large amounts of IL-6, IFNγ, TNFα, GM-CSF, and IL-12 [52]. In MS patients specifically, B-cells secrete increased amounts of the inflammatory cytokines IL-6 [53] and GM-CSF [54] while producing lower levels of the anti-inflammatory cytokine IL-10 [55] compared to healthy controls. Strikingly, elevated IL-6 levels in MS patients are normalized to healthy levels in the blood following B-cell-depletion through rituximab therapy [53]. 

Finally, B-cells can contribute to disease progression through their role as antigen-presenting cells to helper T cells. B-cells are able to process and present auto-antigens captured by their B-cell receptor onto their HLA receptors. This in turn can serve to activate the cognate autoreactive helper T cells via their TCR and additional co-stimulatory molecules such as CD40, CD80, and CD86. One study found that B-cells taken from MS patients were capable of activating helper T cells in the presence of neuro-antigens while B-cells from healthy controls were not [56]. This indicates that B-cells from MS patients can maintain disease by reactivating helper T cells. Additionally, others have found that B-cells from MS patients have elevated expression of HLA-DR, CD40 [57] and CD80 [58]. 

It is clear that B-cells can contribute to disease progression in MS through the three mechanisms of autoantibodies, inflammatory cytokines, and antigen presentation. However, the role of B-cells in MS is complicated because they can also help to protect against disease and inhibit pathology. The most recognized B-cell subset that may protect against MS is the regulatory B-cell (Bregs) population. These B-cells produce large amounts of anti-inflammatory cytokines such as IL-10, IL-35, and TGF-β. There are ample reports in the literature examining how Bregs function and develop [59], but this review will focus on the role of regulatory B-cells in MS. To begin with, several groups have demonstrated that B-cells taken from MS patients produce less IL-10 compared to B-cells taken from healthy controls. These MS B-cells also produce less IL-10 upon stimulation with Cpg [60] or MOG peptide [61]. 

This activity of regulatory B-cells could explain why other B-cell-targeting therapies have not shown efficacy in human trials. The most striking result demonstrating a potential regulatory mechanism of B-cells was from the clinical trial for the drug Atacicept. This trial was stopped early because it resulted in an increase in the relapse rate for the patients [62]. Like anti-CD20 therapies, Atacicept reduces circulating B-cell numbers. However, instead of directly killing cells, Atacicept reduces B-cells by neutralizing BAFF and APRIL, two cytokines that are important for B-cell development, survival and function. It is still not clear why reducing B-cells with anti-CD20 or Atacicept have such disparate clinical trial results. One possible explanation is that certain regulatory B-cell subsets are being targeted in Atacicept treatment, although the mechanism needs to be studied further.

## 4. TFH Cells in Multiple Sclerosis

The presence of autoantibodies in the central nervous system described above indicates that specific B-cell clones are undergoing selection and proliferating. This process of somatic hypermutation and clonal selection of B-cells typically takes place in germinal center-like structures and requires the support of follicular helper T cells. Unfortunately, few studies have looked carefully at the role of follicular T helper cells specifically in MS. One of the seminal reports in the field shows that TFH cells are elevated in the blood of MS patients and this population is positively correlated with the progression of disability [17]. Additionally, the same study found that MS patients have an elevated ratio of CCR6+ Th17-like TFH cells compared to healthy controls. The Th17-like TFH cells are considered the most pathogenic and are the most effective cell type in supporting B-cell activity, particularly antibody production [39]. Importantly, another study has demonstrated that effective treatment with dimethyl fumarate results in a rebalancing of these ratios by decreasing the pathogenic Th17-like TFH cells and increasing the Th2-like TFH cells [63]. It is currently unclear how changes in these occur.

Apart from TFH cells explicitly, there are numerous markers associated with follicular T helper cells which are elevated in patients with MS. One common marker associated with MS is IL-21; the major cytokine produced by follicular T helper cells. Research has shown that progressive patients have increased expression of both IL-21 and IL-21 receptor (IL21-R) in their blood compared to healthy individuals [17]. IL-21 producing T- circulating TFH-like cells affect disease, but it is likely that Th17-like TFH cells can better promote B-cell survival and class-switching, leading to further disease pathology. 

In addition to altered ratios of TFH subsets, patients with MS have also been shown to be deficient in regulatory follicular T helper cells [64]. This regulatory population has recently been shown to play an inhibitory role in many autoimmune disorders and diseases. In patients with MS in particular, these regulatory TFH cells are fewer in number and less functional compared to their healthy counterparts [65].

TFH cells are also present in the lesions of MS patients further supporting their role in the disease [66]. Finally, lowered levels of blood IL-21 levels were correlated with reduced disease activity following treatment with mitoxantrone [17]. 

Other evidence supporting the importance of TFH cells were generated from genome-wide association studies (GWAS) of MS patients. These studies showed that polymorphisms in the TFH genes IL-21 [67], CXCR5 [68], and PD-1 [69] were found to be either diagnostic or prognostic risk factors for MS. 

While TFH cells and molecules associated with this cell population seem to be correlated with increased disease in MS patients (Table 1), it remains unclear how this subset of cells promotes disease. One potential mechanism through which TFH cells can contribute to disease is promoting the inflammatory B-cell activities discussed above. This can be accomplished through their activity in the germinal center and through the production of cytokines. These same processes can occur outside of the germinal center in sites known as ectopic follicles. 

## 5. Ectopic Lymphoid-Like Structures in Multiple Sclerosis

In MS, neuro-inflammation produces cytokines and chemokines that result in immune cell infiltration into the central nervous system. These infiltrating cells are frequently dispersed across the CNS with more concentrated clusters in lesions and areas of active disease based on the concentration of chemoattractant and survival molecules. These clustered populations of infiltrating immune cells can develop a more complex organization as disease progresses. This organization can begin to resemble the structures and arrangement of cells within the follicles found in secondary lymphoid tissues like the spleen or tonsils. A significant population of MS patients (as much as 40% of SPMS patients) [80] can develop these ectopic lymphoid-like structures (ELS) within their CNS tissue, particularly in the meningeal regions. These organized structures consist of B-cell clusters surrounded by T cells and provide an opportunity for germinal center reactions to take place within the site of inflammation. In these structures, B-cells can undergo both somatic hypermutation and class-switching. These B-cells can also differentiate into plasma cells and memory B-cells. As B-cells, particularly those later in development, can promote disease, it is not surprising that the presence of these follicle-like structures in the CNS of progressive MS patients is correlated with faster and more severe disease progression over time [80]. 

Because these ELSs resemble germinal centers in both structure and function, many of the cells and cytokines associated with proper germinal center reactions are also associated with the development of ectopic follicles. Molecules like IL-7, lymphotoxin alpha (LTa), and receptor activator of NF-κB ligand (RANKL) are necessary for the proper development of both germinal centers and ectopic follicles [81]. Additionally, chemokines like CXCL13 and CCL21 are critical to bring the T cells and B-cells together and form the complex arrangement of cells. Interestingly, CXCL13 is the primary ligand for CXCR5, found on TFH cells and B-cells. CXCL13 protein has been shown to be elevated in the blood of MS patients [82] and transcripts are specifically elevated in active lesions compared to inactive lesions [77]. In the brains of patients, these ectopic lymphoid follicles in the CNS are associated with more severe disease including microglia activation and demyelination [80]. Additionally, these ELFs colocalize with demyelinating lesions [83]. 

Although the development of these structures in tissue is not completely understood, there have been many studies looking at the formation of ELSs in many different autoimmune disease models. Through these studies, it has become clear that both TH17 and TFH cells play important roles these ectopic follicles. TH17 cells and the IL-17 they produce are critical to the development of autoreactive germinal centers [84]. Once the follicle is formed, the presence of IL-21 and TFH cells likely support B-cell survival and provide signals necessary for affinity maturation and differentiation within the B-cells.

All of these studies together point to an important role for ectopic lymphoid follicles in promoting inflammation and maintaining an autoreactive cell population. Because TFH cells play a critical role in ELS formation and function, it is possible that the primary mechanism of TFH contribution to MS disease pathology is by acting within these ectopic lymphoid follicles and promoting B-cell activity in the CNS tissue. 

## 6. Experimental Autoimmune Encephalomyelitis (EAE) 

Experimental Autoimmune Encephalomyelitis (EAE) is the most common experimental model of MS in animals. It is a broad term describing many different models of CNS autoimmunity induced in a variety of vertebrates including rats, mice, rabbits, guinea pigs, and non-human primates. This model provides insights into the immune processes that occur in MS patients. It provides an opportunity to examine mechanisms of disease and test experimental therapies. In fact, the use of this model has already led to the development of numerous FDA-approved therapies for MS [85,86,87].

EAE is induced by the injection of an antigen generated from myelin protein, along with an adjuvant and *Bordetella pertussis* toxin. The antigen and adjuvant are sufficient to initiate an autoimmune response to myelin and the pertussis toxin may act like an additional adjuvant or help permeabilize the blood brain barrier (BBB) and allows immune cell infiltration into the CNS. This experimental procedure results in an MS-like disease, symptoms of which include inflammation in the CNS, demyelination of neurons, and ascending paralysis. This paralysis is scored daily in a standard method on a scale of 0 to 5. 

There are two primary models of EAE: active EAE and CD4 T cell adoptive transfer EAE (transfer EAE). Active EAE is initiated by immunization with a myelin antigen. Transfer EAE is induced by transferring activated CD4 T cells from active EAE mice into healthy mice. In transfer EAE, donor T cells are cultured in vitro with myelin antigen and polarizing cytokines promote the differentiation into distinct effector T cell subsets, such as T helper (Th)1 or Th17, before they are injected to recipient mice. EAE is a heterogeneous disease and can present differently depending on the induction method, the myelin antigen used, and the recipient mouse strain [88]. 

### 6.1. Different Roles for B-Cells in Different Types of EAE

Similar to MS, in EAE the role of B-cells is complex and is very much dependent on the type of EAE, and the manner in which it is induced. For example, the type of antigen used for EAE induction can determine whether B-cells are necessary for complete disease development. In mice that lack B-cells, immunization with rodent myelin-oligodendrocyte glycoprotein (MOG) peptide _35–55_, results in normal disease progression. However, immunization with the complete recombinant MOG protein in B-cell-deficient mice results in no disease development [89]. These results point to a critical role for B-cells in the initiation of disease in EAE induce with human MOG antigen.

Further studies have indicated that the human and rodent MOG antigens are processed and presented by different APC populations in the mice. They indicate that dendritic cells are primarily responsible for presenting the rodent MOG peptide while B-cells are more efficient at presenting the whole human MOG protein [90,91]. However, this phenomenon does not entirely explain the lack of disease in whole MOG-immunized B-cells-deficient mice because these mice seem to have similar levels of immune response, as measured by cell activation and proliferation, compared to their B-cell-sufficient counterparts [89]. One possible explanation of these results is that B-cells and dendritic cells process the whole protein differently and present different additional epitopes apart from the obviously encephalomyelitic MOG_35–55_ peptide. However, this needs further research to better understand the mechanism. 

Apart from their potential role in the induction of disease through antigen processing and presentation, B-cells have a complex role to play in the progression of disease once it is induced. In the seminal work by Matsushita et al., it was demonstrated that B-cells can have both pro- and anti-inflammatory effects in rodent MOG-peptide induced EAE [92]. They found that treatment with anti-CD20 treatment could either exacerbate disease if administered before disease was induced or, conversely, it would reduce disease if administered at the first clinical signs of EAE. The authors speculated that this result was because early depletion of B-cells primarily reduced regulatory B-cells in the periphery, while later B-cell depletion was able to target the pathogenic B-cells in the CNS which developed after the disease had time to develop. 

Much of the understanding of the various roles of B-cells in neuro-inflammation comes from studies using mouse models. The three primary mechanisms through which B-cells can contribute to disease progression have also been examined using the EAE model. 

The role of autoantibodies in disease has been extensively examined in the EAE model. The transfer of MOG-specific autoantibodies in mice does not induce any measurable disease and transgenic mice with MOG-specific autoreactive B-cells largely fail to develop spontaneous EAE. However, there is ample evidence that autoantibodies can contribute to and exacerbate existing disease. So, while transfer of autoantibodies does not induce disease, their addition during EAE makes the disease more severe [93]. Similarly, crossing transgenic mice with MOG-specific autoreactive CD4+ T cells with MOG-specific B-cells mice results in mice with a high frequency of spontaneous EAE while each transgenic mouse strain has a low incidence of spontaneous disease [94]. Another study that points to the importance of autoantibodies shows that depleting CD19+ B-cells is more effective than CD20+ depletion because it depletes the antibody-secreting plasma cells [95]. 

B-cells can also contribute to disease progression through the production of inflammatory cytokines, particularly IL-6. The importance of B-cell-derived IL-6 was demonstrated by Barr et al., where they demonstrate that the depletion of B-cells is effective at reducing EAE disease progression through the reduction of B-cells-derived IL-6 [53]. This IL-6 was shown to be a significant stimulant for T cells, particularly Th17 cells. In a different autoimmune model, it was shown that B-cell-derived IL-6 was necessary for mice with SLE to develop spontaneous ectopic lymphoid follicles [96]. 

Finally, it has been shown by two independent research groups that MHCII on B-cells was necessary for fulminant disease development in EAE; a model that has been shown to be B-cell-dependent. Furthermore, they found that disease was only developed when the MHCII-positive B-cells were specific for autoantigens. When they used transgenic mice with B-cell receptors that do not recognize MOG, these mice failed to develop disease as well [97,98]. 

### 6.2. TFH Cells in EAE 

Based on the abundant correlative data between TFH cells and disease in MS patients, research has begun to examine TFH cells in the EAE model in order to better understand their mechanism of action in promoting disease. This recent work has begun to shed light onto the role of TFH cells in neuro-inflammation and has mirrored many of the results found in MS patients (Table 1). 

Similar to human studies, many of the studies in mice examining TFH cells are simply looking at correlative data. For example, one study reported that Laquinomod effectively treated human MOG-induced and the efficacy was correlated with reduced TFH frequency and B-cell aggregates in the CNS [99]. This work suggests that TFH cells are linked with disease progression. Furthermore, our research found that, in the CNS tissue, infiltrating TFH numbers are correlated with B-cell numbers. Additionally, the frequency of TFH cells is in turn correlated with the frequency of class-switched B-cells. This correlation was not observed in CXCR5- CD4+ T cells, indicating that there is a functional relationship between B-cells and TFH cells in particular during EAE disease [75]. 

Peters et al., in their examination of ectopic follicles in Th17-EAE, found that the transferred Th17 cells began to take on a TFH-like phenotype in the CNS tissue during EAE. This includes expression of CXCR5, ICOS, and even BCL6 [74]. Importantly, they also found that the transfer of pathogenic Th17 cells into healthy recipient mice led to the development of ectopic lymphoid follicles, while the transfer of Th1, Th2, and Th9 helper T cells did not. Finally, this formation of ELFs in the CNS of Th17-EAE mice was accompanied by the increased expression of the chemokine CXCL13. They speculate that perhaps this chemokine is playing a critical role in the formation of the follicles in the CNS tissue. 

This relationship between CXCL13 and Th17 was examined in several more recent works. First, in Klimatcheva et al., they found that a CXCL13 neutralizing antibody was protective in Th17-induced EAE but had no effect on Th1-EAE in the relapsing-remitting EAE model in SJL mice [100]. In a challenge with the model antigen NP-KLH, they found that anti-CXCL13 did not significantly affect affinity maturation, but seemed to reduce the size and number of germinal centers in the spleen. This led them to suggest that the antibody was inhibiting cell recruitment to the follicle, leading to the reduction of germinal centers [100]. Our group confirmed that the CXCL13 antibody was also able to protect against Th17-EAE in our C57BL/6 model of disease. We also observed that the antibody had limited effects on B-cell trafficking to the CNS, but instead seemed to affect only the CXCR5+ T cells [75]. This is congruent with previous data that CXCL13^−/−^ mice have no significant change in the number of infiltrating B-cells during active EAE [101]. 

Curiously, we found that although B-cell infiltration is not altered by anti-CXCL13, we found that the efficacy of the antibodies relies on the presence of B-cells. In B-cell-deficient mice, there was no significant effect of anti-CXCL13 despite reduction in the TFH population in the CNS. Therefore, we speculate that anti-CXCL13 is disrupting the TFH population in the CNS, which is in turn disrupting the pro-inflammatory activities of B-cells discussed above [75]. Recently, it was shown that TFH cells are also elevated in active EAE within both the spleen and spinal cord of mice with EAE. These TFH cells led to increased MOG-specific antibodies via an IL21-dependant mechanism. Finally, the transfer of these TFH cells to mice with EAE prolonged the severity of paralysis compared to mice that did not receive an injection of TFH cells [102].

## 7. The TH17-TFH-B Cell Axis in MS and EAE

Th17 CD4+ helper T cells, a unique subset of helper T cell capable of producing IL-17, were discovered in 2005 [103,104]. Around this time, it was demonstrated that IL-23 is a critical cytokine in EAE development [105], and it leads to the development of T cells which produce IL-17 in neuroinflammation [106]. Since these critical discoveries, it has become clear that Th17 cells are an important subset of T cells in the development of EAE. In MS patients, there is evidence of IL17-producing cells infiltrating CNS tissue [107]. These Th17 cells are elevated in the cerebrospinal fluid (CSF) of MS patients during relapses [108]. It was also demonstrated that Th17 cells can traffic to the CNS and infiltrate across the blood brain barrier (BBB) via the choroid plexus [109]. 

Importantly, pathogenic Th17 are producers of IL-21 and are effective B-cell helpers [110]. Similarly, there is significant evidence that B-cells in turn can support the survival and activities of Th17 cells under both normal conditions [111] and in MS disease [56]. Because of this close relationship between these two populations, it is not surprising that a Th17 signature is associated with elevated numbers of B-cells [75,112] and even ectopic lymphoid follicles [74]. 

There is also evidence that Th17 cells provide the inflammatory environment to induce the trafficking of TFH cells into the CNS. We found that myelin-specific TFH cells alone were unable to traffic into the CNS; myelin-specific Th17 cells could traffic into the CNS and lead to a second wave of TFH cells infiltrating the CNS [75]. These data demonstrate that Th17 cells create an inflammatory environment that allow TFH cells to enter the CNS, proliferate, and contribute to the severity of inflammation. One interesting variation of this mechanism in human disease is the Th17-like TFH cells. These cells have the chemokine receptor of Th17 cells as well as the capacity to produce Th17 cytokines. Therefore these TFH cells could, in theory, initiate disease and act in their TFH role as disease develops.

Our data also suggest that the infiltration of TFH cells into the CNS is significantly dependent on CXCL13 because the blockade of CXCL13 through neutralization antibody treatment results in a decrease in the CNS TFH population [75]. Interestingly, this treatment does not alter the infiltration of Th17 or B-cell populations. Therefore, B-cells must be trafficking into the CNS independent of CXCL13 [101]. One possible mechanism is through the chemokine CXCL12 which has been shown to play a role in B-cell trafficking [113] and is also present in MS lesions [76]. 

**Figure 1:**
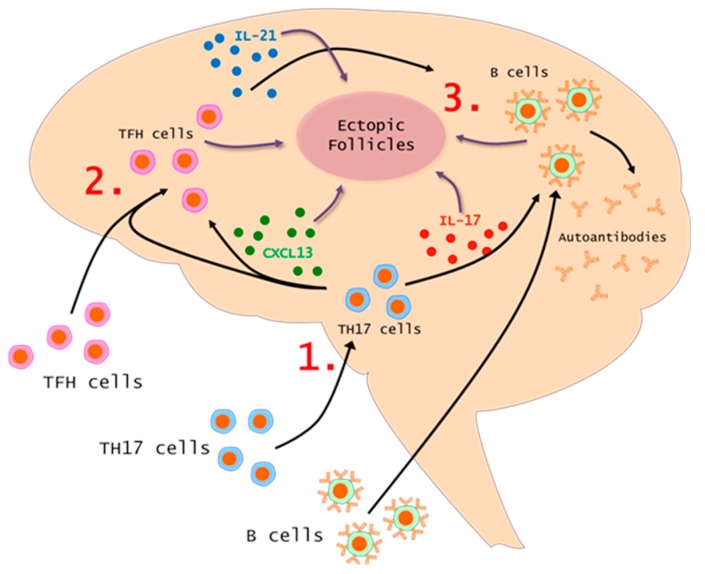
Model of Th17-TFH-B-cell Axis in MS. Th17 cells infiltrate the CNS, secrete IL-17, and induce inflammation. (**1**) Th17 responses drive the production of the chemokine CXCL13 which will facilitate the trafficking of TFH cells into the CNS. (**2**) Once in the CNS, TFH cells secrete IL-21 and other factors to promote ectopic follicles and pathogenic B-cell responses. (**3**). Figure prepared using Motifolio, Toolkit (Motifolio Inc, MD, USA).

These data taken together point to an axis linking pathogenic Th17 cells, TFH cells, and B-cells (Figure 1). This connection is robust because each population supports the survival, proliferation, and activities of the other populations. It is also reinforced by several key cytokines which act on, or are produced by these three cell populations. These cytokines include those discussed throughout this review such as IL-21, CXCL13, and BAFF. This axis is found in many other autoimmune disorders such as SLE, RA, Sjogren’s Syndrome, and neuromyelitis optica (NMO). This further shows how fundamental these relationships are to a pathogenic immune response.

## 8. Conclusions

In the past decade, follicular T helper cells have been shown to be critical mediators of immune responses in many different infection and autoimmune models, and this seems to be consistent in multiple sclerosis. Although the data is primarily correlative in patient samples, it has been consistently shown that TFH cells, their markers, and their products are elevated in MS patients, particularly when disease is elevated. This also goes for Th17 cells and B-cells. Complimentary studies in the mouse model of the disease have shown more clearly that TFH cells are critical mediators of disease activity and particularly inflammatory B-cells activities. Therefore, these cells could be an important clinical target moving forward and could serve as a crucial link within the inflammatory axis consisting of Th17 cells, TFH cells, and B-cells. 

## Figures and Tables

**Table 1:** Follicular T helper cell (TFH) molecules in multiple sclerosis (MS) and experimental autoimmune encephalomyelitis (EAE).

Molecule	Multiple Sclerosis Data	EAE Data	References
**IL-21**	-Increased gene expression in CD4+ cells from blood and lesions -Reduced in blood following treatment (mitoxantrone) -Genetic association with MS (GWAS)	-Decreased EAE in IL-21 knockout mice -Additional IL-21 before EAE increases EAE -IL-21 knockout mice still develop EAE	[17,66,67,70,71,72,73]
**IL-21R**	-Increased gene expression in CD4+ cells from blood and lesions	-IL-21R knockout mice still develop EAE -IL21R knockout mice do not develop spontaneous EAE	[17,72,73]
**CXCR5**	-Increased CXCR5+ cells in blood and lesions -Genetic association with MS (GWAS)	-Increased CXCR5+ cells in central nervous system (CNS) tissue during EAE	[17,68,74,75]
**CXCL13**	-Increased protein in CSF and blood of RRMS patients -Increased gene expression in active lesions vs inactive -Reduced in blood following treatment (Rituximab)	-Increased in CNS tissue during EAE -Blocking CXCL13 is protective in EAE	[74,75,76,77,78]
**ICOS**	-Increased gene expression in CD4+ cells from blood and CSF -Increased ICOS+ cells in blood	-Increased ICOS+ cells in CNS tissue during EAE -Blocking ICOS late is protective in EAE	[17,74,79]

## References

[1] Lassmann H., Bruck W., Lucchinetti C.F. (2007). The immunopathology of multiple sclerosis: An overview. Brain Pathol..

[2] Hafler D.A. (2004). Multiple sclerosis. J. Clin. Investig..

[3] Dendrou C.A., Fugger L., Friese M.A. (2015). Immunopathology of multiple sclerosis. Nat. Rev. Immunol..

[4] Wingerchuk D.M., Carter J.L. (2014). Multiple sclerosis: Current and emerging disease-modifying therapies and treatment strategies. Mayo Clin. Proc..

[5] Sawcer S., Hellenthal G., Pirinen M., Spencer C.C., Patsopoulos N.A., Moutsianas L., Dilthey A., Su Z., Freeman C., Hunt S.E. (2011). Genetic risk and a primary role for cell-mediated immune mechanisms in multiple sclerosis. Nature.

[6] Rangachari M., Kuchroo V.K. (2013). Using EAE to better understand principles of immune function and autoimmune pathology. J. Autoimmun..

[7] Bourdette D., Yadav V. (2008). B-cell depletion with rituximab in relapsing-remitting multiple sclerosis. Curr. Neurol. Neurosci. Rep..

[8] Kappos L., Li D., Calabresi P.A., O’Connor P., Bar-Or A., Barkhof F., Yin M., Leppert D., Glanzman R., Tinbergen J. (2011). Ocrelizumab in relapsing-remitting multiple sclerosis: A phase 2, randomised, placebo-controlled, multicentre trial. Lancet.

[9] Schaerli P., Willimann K., Lang A.B., Lipp M., Loetscher P., Moser B. (2000). CXC chemokine receptor 5 expression defines follicular homing T cells with B cell helper function. J. Exp. Med..

[10] Kim C.H., Rott L.S., Clark-Lewis I., Campbell D.J., Wu L., Butcher E.C. (2001). Subspecialization of CXCR5+ T cells: B helper activity is focused in a germinal center-localized subset of CXCR5+ T cells. J. Exp. Med..

[11] Breitfeld D., Ohl L., Kremmer E., Ellwart J., Sallusto F., Lipp M., Forster R. (2000). Follicular B helper T cells express CXC chemokine receptor 5, localize to B cell follicles, and support immunoglobulin production. J. Exp. Med..

[12] Yu D., Rao S., Tsai L.M., Lee S.K., He Y., Sutcliffe E.L., Srivastava M., Linterman M., Zheng L., Simpson N. (2009). The transcriptional repressor Bcl-6 directs T follicular helper cell lineage commitment. Immunity.

[13] Johnston R.J., Poholek A.C., DiToro D., Yusuf I., Eto D., Barnett B., Dent A.L., Craft J., Crotty S. (2009). Bcl6 and Blimp-1 are reciprocal and antagonistic regulators of T follicular helper cell differentiation. Science.

[14] Nurieva R.I., Chung Y., Martinez G.J., Yang X.O., Tanaka S., Matskevitch T.D., Wang Y.H., Dong C. (2009). Bcl6 mediates the development of T follicular helper cells. Science.

[15] Blanco P., Ueno H., Schmitt N. (2016). T follicular helper (Tfh) cells in lupus: Activation and involvement in SLE pathogenesis. Eur. J. Immunol..

[16] Ma J., Zhu C., Ma B., Tian J., Baidoo S.E., Mao C., Wu W., Chen J., Tong J., Yang M. (2012). Increased frequency of circulating follicular helper T cells in patients with rheumatoid arthritis. Clin. Dev. Immunol..

[17] Romme Christensen J., Bornsen L., Ratzer R., Piehl F., Khademi M., Olsson T., Sorensen P.S., Sellebjerg F. (2013). Systemic inflammation in progressive multiple sclerosis involves follicular T-helper, Th17- and activated B-cells and correlates with progression. PLoS ONE.

[18] Crotty S. (2014). T follicular helper cell differentiation, function, and roles in disease. Immunity.

[19] Crotty S. (2011). Follicular helper CD4 T cells (TFH). Annu. Rev. Immunol..

[20] Vogelzang A., McGuire H.M., Yu D., Sprent J., Mackay C.R., King C. (2008). A fundamental role for interleukin-21 in the generation of T follicular helper cells. Immunity.

[21] Eto D., Lao C., DiToro D., Barnett B., Escobar T.C., Kageyama R., Yusuf I., Crotty S. (2011). IL-21 and IL-6 are critical for different aspects of B cell immunity and redundantly induce optimal follicular helper CD4 T cell (Tfh) differentiation. PLoS ONE.

[22] Choi Y.S., Eto D., Yang J.A., Lao C., Crotty S. (2013). Cutting edge: STAT1 is required for IL-6-mediated Bcl6 induction for early follicular helper cell differentiation. J. Immunol..

[23] Linterman M.A., Beaton L., Yu D., Ramiscal R.R., Srivastava M., Hogan J.J., Verma N.K., Smyth M.J., Rigby R.J., Vinuesa C.G. (2010). IL-21 acts directly on B cells to regulate Bcl-6 expression and germinal center responses. J. Exp. Med..

[24] Poholek A.C., Hansen K., Hernandez S.G., Eto D., Chandele A., Weinstein J.S., Dong X., Odegard J.M., Kaech S.M., Dent A.L. (2010). In vivo regulation of Bcl6 and T follicular helper cell development. J. Immunol..

[25] Fazilleau N., McHeyzer-Williams L.J., Rosen H., McHeyzer-Williams M.G. (2009). The function of follicular helper T cells is regulated by the strength of T cell antigen receptor binding. Nat. Immunol..

[26] Akiba H., Takeda K., Kojima Y., Usui Y., Harada N., Yamazaki T., Ma J., Tezuka K., Yagita H., Okumura K. (2005). The Role of ICOS in the CXCR5+ Follicular B Helper T Cell Maintenance In Vivo. J. Immunol..

[27] Xu H., Li X., Liu D., Li J., Zhang X., Chen X., Hou S., Peng L., Xu C., Liu W. (2013). Follicular T-helper cell recruitment governed by bystander B cells and ICOS-driven motility. Nature.

[28] Qi H., Cannons J.L., Klauschen F., Schwartzberg P.L., Germain R.N. (2008). SAP-controlled T-B cell interactions underlie germinal centre formation. Nature.

[29] Batten M., Ramamoorthi N., Kljavin N.M., Ma C.S., Cox J.H., Dengler H.S., Danilenko D.M., Caplazi P., Wong M., Fulcher D.A. (2010). IL-27 supports germinal center function by enhancing IL-21 production and the function of T follicular helper cells. J. Exp. Med..

[30] Reinhardt R.L., Liang H.E., Locksley R.M. (2009). Cytokine-secreting follicular T cells shape the antibody repertoire. Nat. Immunol..

[31] Goenka R., Matthews A.H., Zhang B., O’Neill P.J., Scholz J.L., Migone T.S., Leonard W.J., Stohl W., Hershberg U., Cancro M.P. (2014). Local BLyS production by T follicular cells mediates retention of high affinity B cells during affinity maturation. J. Exp. Med..

[32] Gu-Trantien C., Migliori E., Buisseret L., de Wind A., Brohee S., Garaud S., Noel G., Dang Chi V.L., Lodewyckx J.N., Naveaux C. (2017). CXCL13-producing TFH cells link immune suppression and adaptive memory in human breast cancer. JCI Insight.

[33] Koncz G., Hueber A.O. (2012). The Fas/CD95 Receptor Regulates the Death of Autoreactive B Cells and the Selection of Antigen-Specific B Cells. Front. Immunol..

[34] Keir M.E., Butte M.J., Freeman G.J., Sharpe A.H. (2008). PD-1 and its ligands in tolerance and immunity. Annu. Rev. Immunol..

[35] Good-Jacobson K.L., Szumilas C.G., Chen L., Sharpe A.H., Tomayko M.M., Shlomchik M.J. (2010). PD-1 regulates germinal center B cell survival and the formation and affinity of long-lived plasma cells. Nat. Immunol..

[36] Ozaki K., Spolski R., Ettinger R., Kim H.P., Wang G., Qi C.F., Hwu P., Shaffer D.J., Akilesh S., Roopenian D.C. (2004). Regulation of B cell differentiation and plasma cell generation by IL-21, a novel inducer of Blimp-1 and Bcl-6. J. Immunol..

[37] Suan D., Krautler N.J., Maag J.L.V., Butt D., Bourne K., Hermes J.R., Avery D.T., Young C., Statham A., Elliott M. (2017). CCR6 Defines Memory B Cell Precursors in Mouse and Human Germinal Centers, Revealing Light-Zone Location and Predominant Low Antigen Affinity. Immunity.

[38] Schaerli P., Loetscher P., Moser B. (2001). Cutting edge: Induction of follicular homing precedes effector Th cell development. J. Immunol..

[39] Morita R., Schmitt N., Bentebibel S.E., Ranganathan R., Bourdery L., Zurawski G., Foucat E., Dullaers M., Oh S., Sabzghabaei N. (2011). Human blood CXCR5(+)CD4(+) T cells are counterparts of T follicular cells and contain specific subsets that differentially support antibody secretion. Immunity.

[40] Chevalier N., Jarrossay D., Ho E., Avery D.T., Ma C.S., Yu D., Sallusto F., Tangye S.G., Mackay C.R. (2011). CXCR5 expressing human central memory CD4 T cells and their relevance for humoral immune responses. J. Immunol..

[41] He J., Tsai L.M., Leong Y.A., Hu X., Ma C.S., Chevalier N., Sun X., Vandenberg K., Rockman S., Ding Y. (2013). Circulating precursor CCR7(lo)PD-1(hi) CXCR5(+) CD4(+) T cells indicate Tfh cell activity and promote antibody responses upon antigen reexposure. Immunity.

[42] Bentebibel S.E., Lopez S., Obermoser G., Schmitt N., Mueller C., Harrod C., Flano E., Mejias A., Albrecht R.A., Blankenship D. (2013). Induction of ICOS+CXCR3+CXCR5+ TH cells correlates with antibody responses to influenza vaccination. Sci. Transl. Med..

[43] Simpson N., Gatenby P.A., Wilson A., Malik S., Fulcher D.A., Tangye S.G., Manku H., Vyse T.J., Roncador G., Huttley G.A. (2010). Expansion of circulating T cells resembling follicular helper T cells is a fixed phenotype that identifies a subset of severe systemic lupus erythematosus. Arthritis Rheum.

[44] Locci M., Havenar-Daughton C., Landais E., Wu J., Kroenke M.A., Arlehamn C.L., Su L.F., Cubas R., Davis M.M., Sette A. (2013). Human circulating PD-1+CXCR3-CXCR5+ memory Tfh cells are highly functional and correlate with broadly neutralizing HIV antibody responses. Immunity.

[45] Boswell K.L., Paris R., Boritz E., Ambrozak D., Yamamoto T., Darko S., Wloka K., Wheatley A., Narpala S., McDermott A. (2014). Loss of circulating CD4 T cells with B cell helper function during chronic HIV infection. PLoS Pathog..

[46] Chung Y., Tanaka S., Chu F., Nurieva R.I., Martinez G.J., Rawal S., Wang Y.H., Lim H., Reynolds J.M., Zhou X.H. (2011). Follicular regulatory T cells expressing Foxp3 and Bcl-6 suppress germinal center reactions. Nat. Med..

[47] Wollenberg I., Agua-Doce A., Hernandez A., Almeida C., Oliveira V.G., Faro J., Graca L. (2011). Regulation of the germinal center reaction by Foxp3+ follicular regulatory T cells. J. Immunol..

[48] Hauser S.L., Waubant E., Arnold D.L., Vollmer T., Antel J., Fox R.J., Bar-Or A., Panzara M., Sarkar N., Agarwal S. (2008). B-cell depletion with rituximab in relapsing-remitting multiple sclerosis. N. Engl. J. Med..

[49] Owens G.P., Bennett J.L., Lassmann H., O’Connor K.C., Ritchie A.M., Shearer A., Lam C., Yu X., Birlea M., DuPree C. (2009). Antibodies produced by clonally expanded plasma cells in multiple sclerosis cerebrospinal fluid. Ann. Neurol..

[50] Keegan M., Konig F., McClelland R., Bruck W., Morales Y., Bitsch A., Panitch H., Lassmann H., Weinshenker B., Rodriguez M. (2005). Relation between humoral pathological changes in multiple sclerosis and response to therapeutic plasma exchange. Lancet.

[51] Heigl F., Hettich R., Arendt R., Durner J., Koehler J., Mauch E. (2013). Immunoadsorption in steroid-refractory multiple sclerosis: Clinical experience in 60 patients. Atheroscler Suppl..

[52] Ayoglu B., Mitsios N., Kockum I., Khademi M., Zandian A., Sjoberg R., Forsstrom B., Bredenberg J., Lima Bomfim I., Holmgren E. (2016). Anoctamin 2 identified as an autoimmune target in multiple sclerosis. Proc. Natl. Acad. Sci. USA.

[53] Barr T.A., Shen P., Brown S., Lampropoulou V., Roch T., Lawrie S., Fan B., O’Connor R.A., Anderton S.M., Bar-Or A. (2012). B cell depletion therapy ameliorates autoimmune disease through ablation of IL-6-producing B cells. J. Exp. Med..

[54] Li R., Rezk A., Miyazaki Y., Hilgenberg E., Touil H., Shen P., Moore C.S., Michel L., Althekair F., Rajasekharan S. (2015). Proinflammatory GM-CSF-producing B cells in multiple sclerosis and B cell depletion therapy. Sci. Transl. Med..

[55] Ireland S.J., Blazek M., Harp C.T., Greenberg B., Frohman E.M., Davis L.S., Monson N.L. (2012). Antibody-independent B cell effector functions in relapsing remitting multiple sclerosis: Clues to increased inflammatory and reduced regulatory B cell capacity. Autoimmunity.

[56] Ireland S.J., Guzman A.A., Frohman E.M., Monson N.L. (2016). B cells from relapsing remitting multiple sclerosis patients support neuro-antigen-specific Th17 responses. J. Neuroimmunol..

[57] Mathias A., Perriard G., Canales M., Soneson C., Delorenzi M., Schluep M., Du Pasquier R.A. (2017). Increased ex vivo antigen presentation profile of B cells in multiple sclerosis. Mult. Scler..

[58] Aung L.L., Balashov K.E. (2015). Decreased Dicer expression is linked to increased expression of co-stimulatory molecule CD80 on B cells in multiple sclerosis. Mult. Scler..

[59] Mauri C., Bosma A. (2012). Immune regulatory function of B cells. Annu. Rev. Immunol..

[60] Bar-Or A., Fawaz L., Fan B., Darlington P.J., Rieger A., Ghorayeb C., Calabresi P.A., Waubant E., Hauser S.L., Zhang J. (2010). Abnormal B-cell cytokine responses a trigger of T-cell-mediated disease in MS?. Ann.Neurol..

[61] Piancone F., Saresella M., Marventano I., La Rosa F., Zoppis M., Agostini S., Longhi R., Caputo D., Mendozzi L., Rovaris M. (2016). B Lymphocytes in Multiple Sclerosis: Bregs and BTLA/CD272 Expressing-CD19+ Lymphocytes Modulate Disease Severity. Sci. Rep..

[62] Kappos L., Hartung H.P., Freedman M.S., Boyko A., Radu E.W., Mikol D.D., Lamarine M., Hyvert Y., Freudensprung U., Plitz T. (2014). Atacicept in multiple sclerosis (ATAMS): A randomised, placebo-controlled, double-blind, phase 2 trial. Lancet Neurol..

[63] Cunill V., Massot M., Clemente A., Calles C., Andreu V., Nunez V., Lopez-Gomez A., Diaz R.M., Jimenez M.L.R., Pons J. (2018). Relapsing-Remitting Multiple Sclerosis Is Characterized by a T Follicular Cell Pro-Inflammatory Shift, Reverted by Dimethyl Fumarate Treatment. Front. Immunol..

[64] Fu W., Liu X., Lin X., Feng H., Sun L., Li S., Chen H., Tang H., Lu L., Jin W. (2018). Deficiency in T follicular regulatory cells promotes autoimmunity. J. Exp. Med..

[65] Dhaeze T., Peelen E., Hombrouck A., Peeters L., Van Wijmeersch B., Lemkens N., Lemkens P., Somers V., Lucas S., Broux B. (2015). Circulating Follicular Regulatory T Cells Are Defective in Multiple Sclerosis. J. Immunol..

[66] Tzartos J.S., Craner M.J., Friese M.A., Jakobsen K.B., Newcombe J., Esiri M.M., Fugger L. (2011). IL-21 and IL-21 receptor expression in lymphocytes and neurons in multiple sclerosis brain. Am. J. Pathol..

[67] Gharibi T., Kazemi T., Aliparasti M.R., Farhoudi M., Almasi S., Dehghanzadeh R., Seyfizadeh N., Babaloo Z. (2015). Investigation of IL-21 gene polymorphisms (rs2221903, rs2055979) in cases with multiple sclerosis of Azerbaijan, Northwest Iran. Am. J. Clin. Exp. Immunol..

[68] Lill C.M., Schjeide B.M., Graetz C., Ban M., Alcina A., Ortiz M.A., Perez J., Damotte V., Booth D., Lopez de Lapuente A. (2013). MANBA, CXCR5, SOX8, RPS6KB1 and ZBTB46 are genetic risk loci for multiple sclerosis. Brain.

[69] Pawlak-Adamska E., Nowak O., Karabon L., Pokryszko-Dragan A., Partyka A., Tomkiewicz A., Ptaszkowski J., Frydecka I., Podemski R., Dybko J. (2017). PD-1 gene polymorphic variation is linked with first symptom of disease and severity of relapsing-remitting form of MS. J. Neuroimmunol..

[70] Korn T., Bettelli E., Gao W., Awasthi A., Jager A., Strom T.B., Oukka M., Kuchroo V.K. (2007). IL-21 initiates an alternative pathway to induce proinflammatory T(H)17 cells. Nature.

[71] Nurieva R., Yang X.O., Martinez G., Zhang Y., Panopoulos A.D., Ma L., Schluns K., Tian Q., Watowich S.S., Jetten A.M. (2007). Essential autocrine regulation by IL-21 in the generation of inflammatory T cells. Nature.

[72] Coquet J.M., Chakravarti S., Smyth M.J., Godfrey D.I. (2008). Cutting edge: IL-21 is not essential for Th17 differentiation or experimental autoimmune encephalomyelitis. J. Immunol..

[73] Lee Y., Mitsdoerffer M., Xiao S., Gu G., Sobel R.A., Kuchroo V.K. (2015). IL-21R signaling is critical for induction of spontaneous experimental autoimmune encephalomyelitis. J. Clin. Investig..

[74] Peters A., Pitcher L.A., Sullivan J.M., Mitsdoerffer M., Acton S.E., Franz B., Wucherpfennig K., Turley S., Carroll M.C., Sobel R.A. (2011). Th17 cells induce ectopic lymphoid follicles in central nervous system tissue inflammation. Immunity.

[75] Quinn J.L., Kumar G., Agasing A., Ko R.M., Axtell R.C. (2018). Role of TFH Cells in Promoting T Helper 17-Induced Neuroinflammation. Front. Immunol..

[76] Krumbholz M., Theil D., Cepok S., Hemmer B., Kivisakk P., Ransohoff R.M., Hofbauer M., Farina C., Derfuss T., Hartle C. (2006). Chemokines in multiple sclerosis: CXCL12 and CXCL13 up-regulation is differentially linked to CNS immune cell recruitment. Brain.

[77] Khademi M., Kockum I., Andersson M.L., Iacobaeus E., Brundin L., Sellebjerg F., Hillert J., Piehl F., Olsson T. (2011). Cerebrospinal fluid CXCL13 in multiple sclerosis: A suggestive prognostic marker for the disease course. Mult. Scler..

[78] Piccio L., Naismith R.T., Trinkaus K., Klein R.S., Parks B.J., Lyons J.A., Cross A.H. (2010). Changes in B- and T-lymphocyte and chemokine levels with rituximab treatment in multiple sclerosis. Arch. Neurol..

[79] Rottman J.B., Smith T., Tonra J.R., Ganley K., Bloom T., Silva R., Pierce B., Gutierrez-Ramos J.C., Ozkaynak E., Coyle A.J. (2001). The costimulatory molecule ICOS plays an important role in the immunopathogenesis of EAE. Nat. Immunol..

[80] Magliozzi R., Howell O., Vora A., Serafini B., Nicholas R., Puopolo M., Reynolds R., Aloisi F. (2007). Meningeal B-cell follicles in secondary progressive multiple sclerosis associate with early onset of disease and severe cortical pathology. Brain.

[81] Jones G.W., Jones S.A. (2016). Ectopic lymphoid follicles: Inducible centres for generating antigen-specific immune responses within tissues. Immunology.

[82] Festa E.D., Hankiewicz K., Kim S., Skurnick J., Wolansky L.J., Cook S.D., Cadavid D. (2009). Serum levels of CXCL13 are elevated in active multiple sclerosis. Mult. Scler..

[83] Howell O.W., Reeves C.A., Nicholas R., Carassiti D., Radotra B., Gentleman S.M., Serafini B., Aloisi F., Roncaroli F., Magliozzi R. (2011). Meningeal inflammation is widespread and linked to cortical pathology in multiple sclerosis. Brain.

[84] Hsu H.C., Yang P., Wang J., Wu Q., Myers R., Chen J., Yi J., Guentert T., Tousson A., Stanus A.L. (2008). Interleukin 17-producing T helper cells and interleukin 17 orchestrate autoreactive germinal center development in autoimmune BXD2 mice. Nat. Immunol..

[85] Johnson K.P., Brooks B.R., Cohen J.A., Ford C.C., Goldstein J., Lisak R.P., Myers L.W., Panitch H.S., Rose J.W., Schiffer R.B. (1995). Copolymer 1 reduces relapse rate and improves disability in relapsing-remitting multiple sclerosis: Results of a phase III multicenter, double-blind placebo-controlled trial. The Copolymer 1 Multiple Sclerosis Study Group. Neurology.

[86] Yu M., Nishiyama A., Trapp B.D., Tuohy V.K. (1996). Interferon-beta inhibits progression of relapsing-remitting experimental autoimmune encephalomyelitis. J. Neuroimmunol..

[87] Ridge S.C., Sloboda A.E., McReynolds R.A., Levine S., Oronsky A.L., Kerwar S.S. (1985). Suppression of experimental allergic encephalomyelitis by mitoxantrone. Clin. Immunol. Immunopathol..

[88] Steinman L., Zamvil S.S. (2005). Virtues and pitfalls of EAE for the development of therapies for multiple sclerosis. Trends. Immunol..

[89] Lyons J.A., San M., Happ M.P., Cross A.H. (1999). B cells are critical to induction of experimental allergic encephalomyelitis by protein but not by a short encephalitogenic peptide. E. J. Immunol..

[90] Constant S., Sant’Angelo D., Pasqualini T., Taylor T., Levin D., Flavell R., Bottomly K. (1995). Peptide and protein antigens require distinct antigen-presenting cell subsets for the priming of CD4+ T cells. J. Immunol..

[91] Constant S., Schweitzer N., West J., Ranney P., Bottomly K. (1995). B lymphocytes can be competent antigen-presenting cells for priming CD4+ T cells to protein antigens in vivo. J. Immunol..

[92] Matsushita T., Yanaba K., Bouaziz J.D., Fujimoto M., Tedder T.F. (2008). Regulatory B cells inhibit EAE initiation in mice while other B cells promote disease progression. J. Clin. Investig..

[93] Linington C., Bradl M., Lassmann H., Brunner C., Vass K. (1988). Augmentation of demyelination in rat acute allergic encephalomyelitis by circulating mouse monoclonal antibodies directed against a myelin/oligodendrocyte glycoprotein. Am. J. Pathol..

[94] Krishnamoorthy G., Lassmann H., Wekerle H., Holz A. (2006). Spontaneous opticospinal encephalomyelitis in a double-transgenic mouse model of autoimmune T cell/B cell cooperation. J. Clin. Investig..

[95] Chen D., Ireland S.J., Davis L.S., Kong X., Stowe A.M., Wang Y., White W.I., Herbst R., Monson N.L. (2016). Autoreactive CD19+CD20- Plasma Cells Contribute to Disease Severity of Experimental Autoimmune Encephalomyelitis. J. Immunol..

[96] Arkatkar T., Du S.W., Jacobs H.M., Dam E.M., Hou B., Buckner J.H., Rawlings D.J., Jackson S.W. (2017). B cell-derived IL-6 initiates spontaneous germinal center formation during systemic autoimmunity. J. Exp. Med..

[97] Molnarfi N., Schulze-Topphoff U., Weber M.S., Patarroyo J.C., Prod’homme T., Varrin-Doyer M., Shetty A., Linington C., Slavin A.J., Hidalgo J. (2013). MHC class II-dependent B cell APC function is required for induction of CNS autoimmunity independent of myelin-specific antibodies. J. Exp. Med..

[98] Parker Harp C.R., Archambault A.S., Sim J., Ferris S.T., Mikesell R.J., Koni P.A., Shimoda M., Linington C., Russell J.H., Wu G.F. (2015). B cell antigen presentation is sufficient to drive neuroinflammation in an animal model of multiple sclerosis. J. Immunol..

[99] Varrin-Doyer M., Pekarek K.L., Spencer C.M., Bernard C.C., Sobel R.A., Cree B.A., Schulze-Topphoff U., Zamvil S.S. (2016). Treatment of spontaneous EAE by laquinimod reduces Tfh, B cell aggregates, and disease progression. Neurol. Neuroimmunol. Neuroinflamm..

[100] Klimatcheva E., Pandina T., Reilly C., Torno S., Bussler H., Scrivens M., Jonason A., Mallow C., Doherty M., Paris M. (2015). CXCL13 antibody for the treatment of autoimmune disorders. BMC Immunol..

[101] Rainey-Barger E.K., Rumble J.M., Lalor S.J., Esen N., Segal B.M., Irani D.N. (2011). The lymphoid chemokine, CXCL13, is dispensable for the initial recruitment of B cells to the acutely inflamed central nervous system. Brain Behav. Immun..

[102] Guo J., Zhao C., Wu F., Tao L., Zhang C., Zhao D., Yang S., Jiang D., Wang J., Sun Y. (2018). T Follicular Helper-Like Cells Are Involved in the Pathogenesis of Experimental Autoimmune Encephalomyelitis. Front. Immunol..

[103] Harrington L.E., Hatton R.D., Mangan P.R., Turner H., Murphy T.L., Murphy K.M., Weaver C.T. (2005). Interleukin 17-producing CD4+ effector T cells develop via a lineage distinct from the T helper type 1 and 2 lineages. Nat. Immunol..

[104] Park H., Li Z., Yang X.O., Chang S.H., Nurieva R., Wang Y.H., Wang Y., Hood L., Zhu Z., Tian Q. (2005). A distinct lineage of CD4 T cells regulates tissue inflammation by producing interleukin 17. Nat. Immunol..

[105] Cua D.J., Sherlock J., Chen Y., Murphy C.A., Joyce B., Seymour B., Lucian L., To W., Kwan S., Churakova T. (2003). Interleukin-23 rather than interleukin-12 is the critical cytokine for autoimmune inflammation of the brain. Nature.

[106] Fitzgerald D.C., Ciric B., Touil T., Harle H., Grammatikopolou J., Das Sarma J., Gran B., Zhang G.X., Rostami A. (2007). Suppressive effect of IL-27 on encephalitogenic Th17 cells and the effector phase of experimental autoimmune encephalomyelitis. J. Immunol..

[107] Tzartos J.S., Friese M.A., Craner M.J., Palace J., Newcombe J., Esiri M.M., Fugger L. (2008). Interleukin-17 production in central nervous system-infiltrating T cells and glial cells is associated with active disease in multiple sclerosis. Am. J. Pathol..

[108] Brucklacher-Waldert V., Stuerner K., Kolster M., Wolthausen J., Tolosa E. (2009). Phenotypical and functional characterization of T helper 17 cells in multiple sclerosis. Brain.

[109] Reboldi A., Coisne C., Baumjohann D., Benvenuto F., Bottinelli D., Lira S., Uccelli A., Lanzavecchia A., Engelhardt B., Sallusto F. (2009). C-C chemokine receptor 6-regulated entry of TH-17 cells into the CNS through the choroid plexus is required for the initiation of EAE. Nat. Immunol..

[110] Mitsdoerffer M., Lee Y., Jager A., Kim H.J., Korn T., Kolls J.K., Cantor H., Bettelli E., Kuchroo V.K. (2010). Proinflammatory T helper type 17 cells are effective B-cell helpers. Proc. Natl. Acad. Sci. USA.

[111] van de Veerdonk F.L., Lauwerys B., Marijnissen R.J., Timmermans K., Di Padova F., Koenders M.I., Gutierrez-Roelens I., Durez P., Netea M.G., van der Meer J.W. (2011). The anti-CD20 antibody rituximab reduces the Th17 cell response. Arthritis Rheum.

[112] Barbosa R.R., Silva S.P., Silva S.L., Melo A.C., Pedro E., Barbosa M.P., Pereira-Santos M.C., Victorino R.M., Sousa A.E. (2011). Primary B-cell deficiencies reveal a link between human IL-17-producing CD4 T-cell homeostasis and B-cell differentiation. PLoS ONE.

[113] Fleige H., Ravens S., Moschovakis G.L., Bolter J., Willenzon S., Sutter G., Haussler S., Kalinke U., Prinz I., Forster R. (2014). IL-17-induced CXCL12 recruits B cells and induces follicle formation in BALT in the absence of differentiated FDCs. J. Exp. Med..

